# Older adult’s longitudinal experiences of household isolation and social distancing during the COVID‐19 pandemic

**DOI:** 10.1111/opn.12459

**Published:** 2022-03-23

**Authors:** Joanne Brooke, Sandra Dunford, Maria Clark

**Affiliations:** ^1^ 1725 Centre of Social Care, Health and Related Research Birmingham City University Birmingham UK; ^2^ Health, Education and Life Sciences Birmingham City University Birmingham UK

**Keywords:** community care, COVID‐19, older people, phenomenology, qualitative study

## Abstract

**Background:**

Due to the global pandemic, governments have enforced household isolation and social distancing to reduce infection and mortality rate. However, the impact of prolonged enforced isolation for older people who are prone to social isolation and loneliness has yet to be understood.

**Objectives:**

A longitudinal study to understand the lived experience of people aged 70 and older, living in England during COVID‐19 restrictions.

**Methods:**

All participants completed five qualitative telephone interviews from 20 April to 7 July 2020. The majority completed individual interviews (*n* = 13), whilst two participants completed these interviews as a couple. Interviews were audio‐recorded, transcribed verbatim and thematic analysis completed from the perspective of hermeneutic phenomenology.

**Results:**

Three themes included (1) engagement and confusion with government restrictions; (2) socialisation through virtual platforms and opportunistic physical social contact; and (3) accessing health care during COVID‐19 restrictions.

**Conclusion:**

Older people are committed to following government restrictions, and government campaigns need to consider the potential impact of placing an emphasis on avoiding healthcare services. Virtual platforms are supportive but not sufficient to reduce social isolation and loneliness of older people. Thus, nurses supporting older people living in the community need to understand these concepts to provide holistic care and support older people's mental and physical health.

**Implications for practice:**

Nurses are ideally placed to support older people to understand the current government restrictions, when to attend acute healthcare services or to engage virtually with healthcare appointments, and to discuss the risks of physically socialising with others.

**What does this research add to existing knowledge in gerontology?**

- This research adds further knowledge regarding older people's use of technology, including virtual platforms, and their engagement with these processes to access health care.
- This research reinforces the importance of virtual platforms to support older people from feeling socially isolated; however, virtual platforms cannot replace the need for the physical presence of another person.

**What are the implications of this new knowledge for nursing care with older people?**

- Nurses are in an optimal position to support older people to understand the current and changing government COVID‐19 restrictions.
- Nurses understanding of the facilitators and barriers experienced by older people to engage with technology and specifically virtual platforms will empower them to support patients to access appropriate care.
- Nurses are in an optimum position to support older people to understand when they need to engage with acute hospital services during the ongoing pandemic.

**How could the findings be used to influence policy, practice, research or education?**

- This research supports the use of virtual platforms to engage with older people for research, education and healthcare services
- Further research is required to understand how modern‐day communities maintain a sense of community spirit, which is inclusive and supports older people during adverse events.
- There are multiple reasons for not accessing healthcare services, further research to understand the reasons for choosing not to access health care, across a range of socio‐demographics could inform nursing practice on appropriate support and guidance.

## 1 INTRODUCTION

The ongoing COVID‐19 pandemic continues to impact populations worldwide; however, the highest mortality rates have occurred within the population over the age of 70. In England and Wales, 142,986 deaths occurred due to COVID‐19 between the 13 March 2020 and the 3 September 2021, of which 117,205 (82%) were people aged 70 or over (Office of National Statistics, 2020, 2021). In response, governments have implemented social distancing and household isolation to reduce the infection rate and deaths due to COVID‐19.

In England, the government implemented national COVID‐19 restrictions on the 23 March 2020, when it became compulsory for people to isolate with and within their households (Public Health England, 2020). Laws were implemented to ensure people only left their households to provide an essential service or obtain essential goods or for daily exercise (Public Health England, 2020). On the 13 May 2020, these restrictions were relaxed to allow people the freedom to leave their households for outdoor recreation. On the 1 June 2020, the restrictions were further relaxed to allow people to meet up with six other people, or with two households outside. The major change to restrictions occurred on the 4 July, when hotels and restaurants re‐opened; however, gatherings of large groups at weddings, funerals or sports events continued to be restricted.

Since March 2020, the government's continued message for people aged 70 and over has been to maintain social distancing (National Health Service, 2021). The global approach of prolonged household isolation and social distancing has impacted negatively on the physical and mental health of older adults, such as increased loneliness, reduced quality of life, higher levels of anxiety and depression, reduced mobility contributing to frailty, cognitive decline, and a higher risk of mortality (Bailey et al., 2021; Cheng et al., 2020; Gustavsson & Beckman, 2020; Robb et al., 2020; Roschel et al., 2020). However, a limitation of these data is the cross‐sectional approach of data collection.

Social isolation and loneliness are separate and distinct concepts, social isolation is a lack of social contact with family, friends or broader social networks, and the lack of involvement in social activities (Valtorta & Hanratty, 2012). Loneliness is a deficit between the actual and desired quality and quantity of social engagement (Victor et al., 2005), which is subjective, and is experienced as a feeling of anxiety and dissatisfaction with connectedness to family members, friends and community (Beaumont, 2013). Social isolation and loneliness have comparative comprehensive negative outcomes for older people, impacting on their psychological and physiological well‐being and their health behaviours (Nicholson, 2012).

Nurses, especially community or primary care nurses, supporting older people living in the community, have a unique opportunity to reach older adults who are socially isolated. Identification of social isolation and loneliness can be supported by a simple and quick screening process, as part of a holistic assessment, empowering nurses to identify patients at risk, and engage in social prescribing (Public Health England, 2019). A broad definition of social prescribing is the referral of a person to non‐clinical services within their local communities, which are usually provided by voluntary organisations, such as exercise classes, support groups, welfare advice and various activity groups/clubs (Younan et al., 2020).

There are different models of social prescribing, although this approach has been implemented in high‐income countries, such as the UK, Canada, America, Australia and New Zealand (Tierney et al., 2020). In England, nurses and other professionals can refer a patient to a link worker, who is connected to local communities (NHS England & NHS Improvement, 2019). The link worker supports patients to focus on what matters most to them and enables them to connect to the most appropriate services, groups and/or activities within their community. This model of social prescribing has been found to support people to feel less lonely, have a purpose, and improve their well‐being and confidence (Foster et al., 2021).

During the restrictions implemented due to the ongoing COVID‐19 pandemic in England, all non‐essential services were closed, which included voluntary community groups and activities. The longitudinal impact of the loss of social engagement with family and friends, community groups and activities, and stay at home legislation on older people during COVID‐19 has yet to be fully explored. A longitudinal exploration of older individuals lived experience as they occurred may identify both negative and positive impacts of household isolation and social distancing, and the application of coping mechanisms and strategies. The identification of coping mechanisms and strategies may empower community or primary care nurses to support older people through the current and ongoing pandemic. Therefore, the aim of this paper was to explore the lived experience of people aged 70 and older living in England during the government restrictions implemented due to COVID‐19 pandemic (23 March–4 July 2020).

## 2 METHODS

### 2.1 Design

A longitudinal hermeneutic phenomenological design was applied. This philosophical approach was informed by the work commenced by Heidegger (1962) and analysed by Gadamer (1989) and reported by Cohen et al. (2000). Gadamer (1989) was interested in how people understood their lived experience of a phenomena in their world, and it is this understanding that is important and not the phenomena itself. The understanding of a lived experience is bound within interpretation, which is an evolving process, and the interpretation continues through a dialectical interaction with the interpreter, which is in this case the researcher (Laverty, 2003).

The design of this study supported the exploration of older people's lived experience and their understanding of household isolation and social distancing during the COVID‐19 pandemic. The longitudinal approach was essential to explore participant's different experiences and understanding as they occurred over time and as they adjusted to the continuation of government COVID‐19 restrictions.

### 2.2 Setting

Older people living within their own households in England during the COVID‐19 restrictions implemented by the government from 23 March to 4 July 2020.

### 2.3 Recruitment

Due to the current pandemic and the need to protect participants and maintain social distancing, recruitment occurred virtually. Recruitment occurred through the dissemination of a flyer by staff within the Faculty of Health Education and Life Science at Birmingham City University on their neighbourhood groups, and Apps such as Nextdoor App, and Twitter. Potential participants, anyone aged 70 or older living at home, contacted the research team by telephone or email if they were interested in participating in the study.

### 2.4 Participants

A baseline interview was completed with participants before the commencement of this study, to capture retrospective information on their everyday lives prior to the COVID‐19 pandemic, data from this baseline interview have previously been published (Brooke & Clark, 2020). All participants lived in houses with gardens in England (*n* = 15) and lived with either their spouse (*n* = 8), alone (*n* = 5), with their grown‐up child (*n* = 1), or had moved in with a grown‐up child and their family for the duration of the pandemic (*n* = 1). The ethnicity of participants was not collected, and no direct questions exploring ethnicity were included in this study; however, participants described their ethnicity when discussing their past lives or with regards to global events, such as Black Lives Matter, one participant explicitly explored her and her husband's heritage to support my understanding of her experiences during the war, which she related to her experiences of lockdown. Therefore, participants self‐identified as Black Caribbean (*n* = 1), European (*n* = 1) and White English (*n* = 13). Participant's age ranged from 70 to 89 with a mean age of 77 years (Table 1).

**TABLE 1:** Overview of participants

Code name	Age	Living circumstances
Barbara	83	Lives alone
Carole	70	Lives alone
Edith	86	Lives alone
Freda	89	Lives alone
Hilda	71	Lives alone
Jessica	70	Lives with husband
Katherine	75	Lives with husband
Louise	83	Lives with daughter
Martha	75	Lives with husband
Peter and Rosemary	75 and 74	Husband and wife living together
Stephen	84	Lives with daughter
Trevor	72	Lives with wife
Vincent	72	Lives with wife
Walter	71	Lives with wife

Note

All participants lived in a private house with a garden. All names are pseudonyms to protect participant's identities.

### 2.5 Data collection

Five semi‐structured telephone interviews, which were conducted between 20 April 2020 and the 10 July 2020, refer to Table 2 (*n* = 108). The semi‐structure interview guide was developed from the concept of social loneliness (Courtin & Knapp, 2017), and reflected questions from measurements of loneliness, social networks and support (Berkman & Syme, 1979; de Jong Gierveld, 1987; Powers et al., 2004; Russell, 1996), and included open questions to allow each participant to discuss aspects of the pandemic, which was important to them.

**TABLE 2:** Interview schedule

Interview	Dates completed
Baseline	6th–15th April 2020
1	20th–29th April 2020
2	4th–13th May 2020
3	18th–27th May 2020
4	1st–10th June 2020
5	29th June–7th July 2020

Note

Important dates: 23 March 2020, the UK government introduces social distancing measures due to COVID‐19.

Telephone interviews were necessary due to the pandemic to ensure the safety of participants and the researcher. The benefit of telephone interviews included the distribution of power between the participant and the researcher, decreased social pressure to provide socially acceptable answers, whist providing a greater level of anonymity, allowing participants to discuss their feelings and their experiences (Holt, 2010; Vogl, 2013). However, the disadvantage of telephone interviews included a potential loss of visual nonverbal cues such as body language, which support communication (Novick, 2008; Stephens, 2007). The disadvantages were addressed by the adherence to the recommendations developed by Farooq and De Villiers (2017) on completing telephone interviews, including how to support and build rapport with participants, and communicating without visual cues.

Of the five interviews, four interviews were performed at 2‐weekly intervals and the final interview occurred after a 4‐week interval. Interviews were conducted at 2‐weekly intervals during the peak of the pandemic, and the last interview occurred after the national lockdown restrictions had begun to be relaxed. Individual interviews were conducted with 13 participants, whilst two participants completed the interviews as a couple. All interviews were facilitated by the first author, audio‐recorded and transcribed verbatim. Each interview lasted between 15 and 45 min. The verification of the information obtained within each interview (apart from the last interview) was sought at the beginning of the next interview, by the researcher recapping information provided. This allowed participants to comment on their previous interview and a reminder of the information they had discussed.

### 2.6 Data analysis

Thematic analysis, as described by Braun and Clarke (2006), was completed to explore the lived experience of older people living through social isolation due to the COVID‐19 pandemic. Due to the longitudinal nature of this study, each participant's transcripts were analysed in chronological order, supporting the understanding of their lived experience at each time point and how these experiences developed and changed over time. The trustworthiness and credibility of this process were supported by the simultaneous analysis of the data by two of the authors. Furthermore, both authors applied a transparent reflexive and iterative process, which included the documentation of their thoughts, insights and reasons for each decision within the six phases of the analysis process. All data analysis was conducted by both authors by hand.

Both authors (1) read and re‐read the transcripts to become familiar with the data; (2) developed a list of initial codes identified from the data; and (3) identified which codes could be grouped together to develop a theme. Following the completion of these three stages, all three authors discussed the identified themes and relevant codes, and differences and discrepancies were discussed, until agreement was reached. Then, the same two authors worked together to complete the remaining three stages of thematic analysis: (4) themes were reviewed by returning to the data to ensure the amended themes captured the essence of the data; (5) each theme was defined and conceptualised; and (6) the writing of each theme with direct quotes from the participants.

### 2.7 Ethical considerations

Ethical approval for the study was obtained from the Birmingham City University Ethics Committee (6290/Am/2020/Apr/HELS FAEC). All potential participants received a participant information sheet and were able discuss any concerns or questions with the first author, prior to providing oral audio‐recorded consent. On commencing each interview, participants were reminded that their contribution was voluntary, and asked if they wished to continue, and if participants verbally agreed, they were reminded the interview would be audio‐recorded. Participants did not receive any financial incentives.

## 3 FINDINGS

Three themes were identified from the data, which included: (1) engagement and confusion with government restrictions; (2) socialisation via virtual platforms and opportunistic physical social contact; and (3) accessing health care during COVID‐19 restrictions.

### 3.1 Engagement and confusion with government restrictions

During the first month of restrictions, participants avidly followed the government briefings and adhered strictly, although sometimes obsessively to recommendations, such as hand washing. Participants also implemented further cleaning, disinfecting and quarantine routines, due to the need to feel safe and protected in their home environments. Although, these were identified as time consuming, possibly not always necessary, and disruptive to daily routines, ‘I bought some Alcolado, we use this in the Caribbean, it has hypersensitive alcohol in it, and I wash my face in that before I go out, so if anything comes towards me hopefully the alcohol will repel it’. (Carole). ‘I am sterilising the doorbell, letter box and handle, when I have deliveries, I unpack outside. I am a bit neurotic as I also bleach the milk bottles!’ (Barbara). ‘I place all deliveries or shopping in a quarantine box for 72 h, it is a performance’ (Martha).

When the government restrictions were relaxed on the 13 May and then on the 1 June 2020, participants found the new restrictions confusing and difficult to understand, especially the number of people they could meet from different households, this led to feelings of uncertainty and concern that they were no longer safe. Participants also identified this confusion as distressing, as they felt the need to follow the rules, ‘So, I’m getting a little bit confused on what is allowable, and what is not advised, and I do like to play it by the book’ (Katherine). ‘I am getting muddled, when this all started I knew the rules, but now I am not clear… this doesn't sit well with me, because I am a rules girl, you tell me the rules and I will stick to them’ (Hilda). Due to this confusion participants began to complete their own risk analysis, identifying which restrictions they felt would maintain their safety. Risk assessments were informed by information from reliable sources, such as the World Health Organisation.

A risk identified by participants was mixing with other people in unfamiliar places, ‘I am very careful into which environment I go, and I have not been into the shopping centre at all, I have got into the routine of click and collect and not even going to the shops, I am quite happy with that, I am safe’ (Edith). A further risk identified was the belief the COVID‐19 virus would still be present, even when restrictions were stopped, ‘We won't be going to the pub, the cinema, or any gatherings in doors, we might do something outdoors, but we won't be going into any cities’ (Martha). However, a risk analysis may also support participants to break the restrictions, ‘… my grandson's birthday, we broke the rules, because two households joined us, but we have all been in lockdown, so how on earth could they possibly pose a threat to us, they came in cars, they came in the back gate, they hand sanitised, common sense says they proposed no threat’ (Martha).

During the restrictions due to COVID‐19 participants adhered to the guidance, which they felt provided them with structure, and safety. However, when the government restrictions were relaxed participants found these messages confusing, and they felt the need to complete their own risk analysis to understand which restrictions they would continue and were necessary to maintain their own safety.

### 3.2 Socialisation through virtual platforms and opportunistic physical social contact

During the beginning of the government restrictions, participants acknowledged an increase in communication, either by phone or a virtual platform, with friends, family and old acquaintances. Communication via virtual platforms was found to be supportive, ‘Facetime has helped me a lot, especially with my family, I feel a lot better seeing their faces’ (Barbara). Most of the participants were familiar with at least one virtual online platform, which quickly became an important and essential method of communicating and socialising with friends and family, ‘We (Walter and his wife) are using social media a lot more, there is a steady flow of funny videos and enough coming in from friends and family. So, I don't feel completely isolated’. Participants also continued to virtually engage in their previous activities and clubs, ‘I go to Pilates, and there are loads of ladies, and we have a coffee and a good chat afterwards, now we have set up a chat group on Tuesday mornings, where we have our coffee and chat’ (Katherine).

During the second and third months of enforced restrictions, participants entered a development phase, as they began to develop different methods to enable them to engage with friends and family members at a deeper level, ‘We have got used to Zoom, I was speaking to the grandchildren and they were just making silly noises and running off… now I do half an hour with them, where we play games’. (Jessica). ‘We are doing an evening out not going out, over Skype, with two other couples, and the last one we did was quite successful’ (Walter). An important element of socialising virtually was to remain involved and connected to the younger members of the family, ‘On our Zoom there is about 14 of us … the youngsters all chat to each other, and the oldies just sit and listen, and we get to know what is going on in their lives’ (Carole).

However, participants also demonstrated the need for physical social contact to prevent feeling isolated, this was achieved through opportunistic physical social contact, which occurred when participants completed essential shopping or their daily exercise, ‘I walk the dog, and you pass maybe 10 people with a dog, and you have a brief conversation with them at a distance, which is good’ (Louise). One participant discussed the impromptu invitations over the garden fence with her neighbour ‘I have a new neighbour, who invited me to sit in her garden, I have been there three times now, and that is so nice, just to sit and chat’ (Freda).

Opportunistic physical social contact became more prominent as the restrictions were relaxed on the 1 June 2020, and people could meet outside whilst social distancing. The relaxation of the restrictions supported participants to continue to try to abide by the restrictions, whilst engaging in physical social contact ‘I now have a stool at the front door, and people come and sit on the wall and we have a chat, that is lovely’. (Barbara), and ‘I live in a close (small no through road), and the other day three of us (neighbours) had a distant chat, we just saw each other, we then bought our coffees and chairs out, and just had a chat, and it was really nice, we are going to this again!’ (Martha).

During the government restrictions, participants discussed the need for a developmental phase towards virtual platforms to remain connected and involved with their family members and friends. Virtual platforms became more important and essential to support meaningful socialisation, and supported participants to continue socialising with those with shared interests. However, participants also engaged in actively seeking opportunist physical social contact, throughout the government restrictions, as the physical presence of another person was highly valued.

### 3.3 Accessing health care during COVID‐19 restrictions

The need to access healthcare services during the current and ongoing pandemic was a concern to all participants, due to their fear of coming into contact with the COVID‐19 virus. Some participants avoided attending the Emergency Department (ED), even when the need arose. Vincent fell from a ladder whilst cleaning out the guttering on his roof, ‘I decided it wasn't something terrible terrible, and I didn't want to go to hospital as they have COVID patients, so I thought no, just keep away from more trouble’. However, a fortnight later Vincent was still ‘hobbling around with a stiff ankle’, and a month later ‘I have my mobility back in the ankle now, one leg is slightly fatter than the other, but it is a normal colour again’. These comments suggest attending an Emergency Department or Walk in Centre was the most appropriate course of action for Vincent's injury.

Participants who attended the ED described their distress due to being on their own as well as the fear of coming into contact with the COVID‐19 virus. For example, Jessica fell whilst out for a walk and was driven to ED by her friend ‘it was OK really, although you can't go in with anybody, and you felt on your own because you are not well, I did have to go up as I was waiting to be seen, to sort of say can I have some pain killers please’ (Jessica). Although the hospital visit was necessary, Jessica was still concerned regarding the chance of coming into contact with the COVID‐19 virus and the need to protect her friends, ‘I asked my daughter to pick me up, as I don't think it is fair to ask someone to pick me up from a hospital setting. I know I would have my mask on and the window down, but my daughter works here and has already had COVID’.

When participants did engage with healthcare services, their concerns were minimised, though they still described these visits as unnerving due to the emptiness of the hospital, ‘There were no people there, because you have to arrive at your appointment and wait in the car, it is very eerie really, but I must admit I got good attention, but it was all very unnatural’ (Barbara). However, primary care was discussed by all participants in a positive manner, they believed their GPs had become more efficient during the pandemic, and focused on maintaining patients safety, ‘I am on medication, which needs liver and kidney function tests to be completed periodically, and so that is what they did, the phlebotomist walked up the drive and stopped and put all her paraphernalia on, mask, googles, gloves, aprons, I was quite impressed really’ (Martha).

Simultaneously, participants expressed concern due to the cancellation of ongoing investigations, and these included appointments with ophthalmologists and cardiologists. A further concern discussed by participants was the possibility of needing dental treatment during the restrictions, ‘I think I am brewing an abscess on my tooth, my dentist gave me an emergency number and they couriered antibiotics to me, my concern is that our dentist is not doing any work at all’ (Rosemary). In a later interview, Rosemary reported the antibiotics appeared to have worked, although the underlying problem still needed treating.

Participants actively avoided acute healthcare settings to reduce their chances of coming into contact with the COVID‐19 virus. When participants did attend hospital appointments, their fears and anxieties were acknowledged to be unfounded, although their experiences were strange and unnerving, mainly due to being alone and the emptiness of the hospital. Simultaneously, participants were concerned for their health due to cancellations of ongoing investigations, and the inability to access dental care. However, due to the approach of primary care and GP surgeries, with a focus on maintaining patient's safety, these services were praised, and seen as being supportive and more efficient.

## 4 DISCUSSION

The longitudinal exploration of the lived experience of older adults during the COVID‐19 restrictions, which commenced on 23 March 2020 in England, identified three themes that developed over time, including fluctuating engagement and confusion with government restrictions, and the need to complete individual risk assessments to remain safe. Socialisation through virtual platforms, which was supportive but not sufficient, so participants engaged in opportunistic physical social contact. The avoidance and unnerving experiences of accessing acute health care, due to the concern of coming into contact with COVID‐19 and the emptiness of hospitals.

### 4.1 Engagement and confusion with government restrictions

The commitment to government COVID‐19 restrictions at the beginning of the pandemic was demonstrated by all participants within this study. This is comparative with the findings that women, older adults and those more vulnerable to be seriously ill are more likely to adhere to government guidance (Armitage et al., 2021; Carlucci et al., 2020). A socio‐ecological model has been applied to explore adherence to government restrictions during the ongoing COVID‐19 pandemic (Coroiu et al., 2020), of which findings were similar to the current study, as participants adhered to government restrictions at an individual‐level due to the need to keep safe, and at the interpersonal level due to a sense of responsibility to the community.

However, dissimilar to the current study, distrust in the government was identified as a reason for non‐adherence to government restrictions at the individual‐level (Coroiu et al., 2020), whereas non‐adherence in the current study was due to confusion regarding current restrictions. The concept of confidence in the government has also been identified to increase adherence to government restrictions (Wright et al., 2021). However, the results from this study suggest participants may have confidence in the government, but when the messages disseminated are confusing this will affect an individual's adherence to restrictions. A final factor which influenced non‐adherence to government restrictions at an interpersonal level, which was identified across both studies, was the need for physical socialisation (Coroiu et al., 2020).

A unique finding of the current study was the completion of a personal risk analysis by participants to understand the risk they were taking when they chose to socialise with friends or family to avoid feelings of social isolation and loneliness. Personal risk assessments were completed after searching for relevant information and assessing the extent to which the individuals they were visiting adhered to government restrictions. Our study and previous studies highlight the fact older adults may have different reasons for completing a risk analysis and not adhering to government restrictions (Coroiu et al., 2020; Wright et al., 2021). Primary care and community nurses, whilst practising person‐centred care, have the potential to understand their patient's rationale behind personal risk assessments and provide relevant and effective support and guidance. This approach may support older people's adherence to restrictions and prevent the possibility of placing themselves at risk.

### 4.2 Socialisation through virtual platforms and opportunistic physical social contact

**FIGURE 1:**
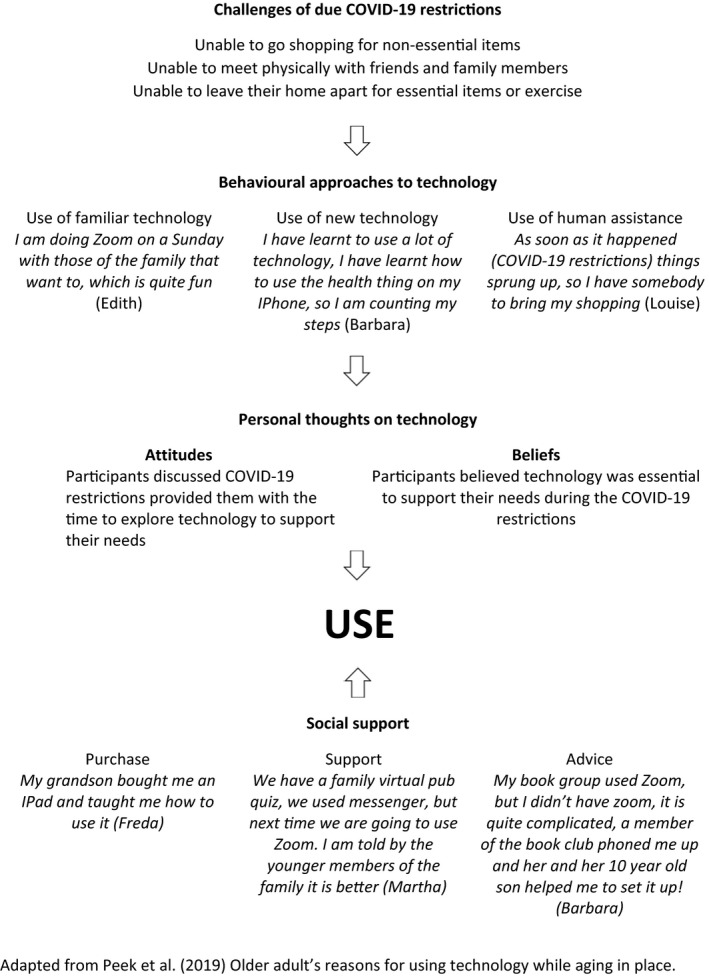
Concept model of older adults using technology during COVID‐19 restrictions

The need to maintain social connections with friends and families by participants was evident within this study from the beginning of the government restrictions, which increased over time, and was only partially maintained through engagement with virtual platforms. A model developed by Peek et al. (2016) identifies six factors which influence the use of technology by older people, which is comparable to the findings of the current study (refer to Figure 1). The first factor, challenges of independent living, was similarly identified in the current study due to the government restrictions and the need for participants to re‐organise their lives through technology, such as online shopping. The second factor identifies four behavioural approaches to technology, of which our participants applied three to meet their new needs, including an increase in their use of familiar virtual platforms, engagement with and use of new virtual platforms, and engaging in opportunistic physical social contact. The last behavioural approach, avoidance of technology, was not identified in the current study.

The third factor of social network (Peek et al., 2016) was identified by all participants in the current study, as they discussed how their children/grandchildren purchased iPads, and supported them in joining virtual platforms, creating profiles, and connecting these to family members and friends. An important element for the participants was to remain engaged and connected with younger members of the family and the ability to follow their posts, which has also been identified prior to the current pandemic (Jung et al., 2017). The fourth factor of personal thoughts on technology identifies attitudes (need, interest, willingness to invest) and beliefs (properties, consequences, proficiency) (Peek et al., 2016). Participants in the current study had the need and time to invest in exploring virtual platforms to support their social needs, which they also believed to be a necessity to support their mental well‐being. The fifth and sixth factors of the model developed by Peek et al. (2016) include organisations and physical environments, which were not overtly identified in this study, although further research explicitly exploring these final factors is required. However, the first four factors were identified in the current study, and are important for all healthcare professionals, especially nurses, as these factors are structured and easily identifiable during a patient visit, providing a structured approach for healthcare professionals to support patients to engage with friends, family, and more importantly virtual healthcare services, both now and beyond the pandemic.

The unique finding from this study, which did not include technology, was the emergence of opportunistic physical social contact, which was essential for participants to meet their social needs and involved participants actively seeking opportunities to socialise outside of their house. Participants benefited from opportunistic physical social contact through connection with their local communities. All participants greatly valued the community in which they lived, and described a community spirit, although they could not always explain components that constructed such a positive community. Participants valued being connected to their local community, which is essential as people who are socially connected live longer and have a lower risk of developing somatic diseases, including heart disease and cancer (Holt‐Lunstad et al., 2010; Miller et al., 2009). Participants in this study had the ability to make opportunistic, and deliberate social connections within their community. Primary care and community nurses can foster the same ability for more isolated patients by having a working knowledge of local community resources and/or engaging in social prescribing (Baileys et al., 2018).

### 4.3 Accessing health care during COVID‐19 restrictions

Participants in the current study were fearful and anxious should the need arise to access acute health care during the COVID‐19 pandemic, due to their belief of an increased risk of coming into contact with the COVID‐19 virus. This is comparative with other studies and across age groups, a European study also identified people were scared to access hospital emergency departments (49%), hospital‐based specialists (42%), dental practice (42%), ambulances (39%), GP practices (39%), but also pharmacies (24%) due to the fear of contracting COVID‐19 (Incisive Health, 2020).

An element that has also been identified in the UK and within this study was the belief of avoiding NHS services to reduce the burden on these services during the pandemic. This may have been influenced in the UK by the government's slogan and mantra of ‘Stay Home, Protect the NHS, Save Lives’, which has been acknowledged as possibly being too successful (Hope & Dixon, 2020). Similarly in the United States, where public health messages focused on strong social distancing and enforcing restrictions, leading to a fear of seeking health services and fewer acute admissions for heart attacks and strokes (Malina, 2020).

However, the impact of the avoidance of healthcare services has yet to be explored and understood for older people. A study performed in South Korea found the prevalence of health service avoidance was higher in certain sociodemographic groups, particularly related to age and residential area characteristics (Lee & You, 2021). Primary care and community nurses may be in an optimum position to support older people to understand when they need to engage with acute hospital services and explain how the need and benefits outweigh the risks. Further research into the reasons for health service avoidance in the UK would support primary care and community nurses to offer relevant and effective guidance.

A unique finding of this study was the positive, although sometimes unnerving experience of engaging with healthcare services during the pandemic, especially the organisation of participants local GP practices. The changes to primary care consultations for older people during COVID‐19 have been explored across GP practices in Oxford, which identified telephone and video consultations doubled for older people at the beginning of the pandemic, although face‐to‐face consultations fell by 64.2% and home visits by 62% (Joy et al., 2020). This is supported by participants in the current study as they discussed how their GP practices had contacted them by phone, although they were asked if they needed any support or their prescriptions delivered. Participants within the current study also appreciated telephone and video consultations with their GPs as they felt this was a good process to seek medical advice, whilst keeping themselves safe.

Whilst virtual and telephone access to primary and secondary services has been available for some time, its use has increased during the current pandemic, allowing the continuation of services. Some acute services are also beginning a shift towards adopting virtual triage for specific ailments, such as fractures (Havenhand et al., 2021). Primary care and community nurses are ideally placed to ensure older adults in their communities are informed of available ways to access services at reduced risk.

### 4.4 Limitations and strengths

The method of recruitment is a limitation, which occurred mainly through virtual platforms, and therefore, it could be expected that most of the participants would be familiar with at least one virtual platform. The second limitation is the possibility participants may not be representative of older people who do not have access to a smart phone, iPad, or an outside space of their own. A further limitation occurred due to the method implemented, which was essential for the hermeneutic phenomenological approach, which was the necessary breadth of the interviews to enable an exploration of the lived experience of restrictions during the COVID‐19 pandemic. However, the phenomenological approach was also a strength of this study, supporting an exploration of the lived experience of older people during the restrictions in place due to COVID‐19 and the identification of novel information.

## 5 CONCLUSION

Older people are committed to following government restrictions when these are clearly disseminated and include a focus on how to maintain their individual safety and the safety of their community. A lesson learnt is not to over emphasise the need to avoid healthcare services, to ensure the continuity of care, and emergency treatment when necessary. Social distancing and household isolation are essential elements of the government restrictions to reduce the infection rate of COVID‐19. However, this increases the risk of social isolation and loneliness amongst older people, and needs to be recognised, addressed and interventions implemented. Virtual platforms for the use of communication with friends and family members are supportive for short periods of time, but not sufficient during restrictions that last longer than a couple of weeks. Therefore, it is important to understand the need for social distancing and how older people can engage safely with friends and family members during the pandemic, which may reduce the need and risk of seeking opportunities for physical social contact. Voluntary and third sector virtual and telephone services are available and should be offered as social support systems. Social prescribers have knowledge of local services and can act as a resource for nurses on available virtual and telephone services which may help to sustain older adults during longer periods of social isolation.

## CONFLICTS OF INTEREST

The authors declare that they have no conflict of interest.

## AUTHOR CONTRIBUTION

All authors contributed to the development of the themes through continuous thematic analysis and discussions, and the development and writing of this paper.

## Data Availability

Author elects to not share data.
